# The Role of Scottish Languages and Dialects in Integrating International Medical Graduates Into the National Health Service: Navigating Linguistic Barriers

**DOI:** 10.7759/cureus.73816

**Published:** 2024-11-16

**Authors:** Usama Al-Kamali, Goran Zangana, Matheel Al-Rawas

**Affiliations:** 1 Unit of Acute and General Medicine, Royal Infirmary of Edinburgh, Edinburgh, GBR; 2 Prosthodontics, School of Dental Sciences, Universiti Sains Malaysia, Kota Bharu, MYS

**Keywords:** clinical practice, cross-cultural communication, international medical graduates, language barriers in healthcare, national health service, scottish dialects, scottish nhs integration

## Abstract

The United Kingdom, particularly Scotland, is a key destination for international medical graduates (IMGs), who now make up a substantial part of the National Health Service (NHS) workforce. These IMGs encounter several challenges when integrating into the NHS, with language barriers being especially significant. Although many IMGs are educated in English, they frequently struggle with the intricacies of Scottish languages and dialects, which are vital for good patient care. This review examines Scotland's linguistic environment, focusing on the roles of Gaelic and Scots languages in cultural distinctiveness and patient communication. By means of a literature review and focus group interviews with IMGs, the authors ascertained commonly utilised Scottish colloquialisms and their connotations, highlighting their importance in clinical contexts. The findings indicate that comprehending such colloquialisms can greatly improve doctor-patient communication, decrease misunderstandings, and enhance health outcomes. The article advocates for the formulation of formal training programs to better equip IMGs for the linguistic challenges they will encounter, thus improving their assimilation into the NHS and enhancing patient care. While the Scottish Government’s efforts to support international recruitment and workforce assimilation have been exemplary, there remains a pressing need for targeted language orientation to close the communication gap and warrant high-quality healthcare delivery.

## Introduction and background

The United Kingdom (UK) is a preferred destination for internationally trained doctors due to its high demand for healthcare workers and favourable work environment [1]. Notably, in 2021, 31.9% of doctors and 17.9% of nurses in the UK were trained abroad, according to the National Health Service (NHS) workforce data [2]. This influx is critical to supporting the UK’s healthcare services, as a significant number of these professionals come from outside the European Economic Area (EEA) [3].

In Scotland specifically, the Scottish NHS has also experienced substantial growth in the number of international medical graduates (IMGs). While specific data for Scotland are limited, the overall UK trend indicates that more than 30% of the UK’s medical workforce now consists of IMGs. This alignment suggests similar patterns of reliance on IMGs across the UK’s constituent countries, including Scotland [3].

The phrase "NHS's multidisciplinary, non-hierarchical team structure" refers to a specific way that healthcare teams operate in the United Kingdom's NHS. In this system, healthcare professionals from various specialities, such as doctors, nurses, pharmacists, and physiotherapists, work collaboratively in a "multidisciplinary" team. This means that instead of one single type of professional handling a patient's care, multiple experts with different skill sets and perspectives come together to provide a holistic approach. This approach is intended to ensure that patient care is comprehensive and considers a wide range of medical, physical, and emotional needs [2].

The "non-hierarchical" aspect indicates that, within these teams, there is less emphasis on traditional power hierarchies, meaning that decisions are not solely made by doctors or senior staff. Instead, all team members, regardless of their role or seniority, are encouraged to share their insights and contribute to decisions about patient care. This collaborative method can be challenging for IMGs if they are used to a more hierarchical system where doctors or other senior members make most decisions and other professionals have more limited input. Adjusting to this structure requires adapting to a work environment where everyone’s voice is valued equally, and communication and teamwork are essential [3].

IMGs encounter numerous obstacles, including language difficulties, cultural adaptation, and academic performance. Particularly, adapting to the NHS's multidisciplinary, non-hierarchical team structure is vital. Language challenges, including regional accents, can hinder integration into clinical settings [4]. Language barriers, particularly understanding regional accents like Scottish dialects, have been identified as a critical challenge for IMGs. The impact of these barriers on clinical communication and patient care is significant, with measures such as local language training and improved communication tools being implemented [5]. Although many IMGs receive their medical training in English, a considerable number are educated in other languages, further complicating their ability to navigate Scotland's linguistic intricacies [3]. In spite of the importance of these challenges, there are presently no formal induction programs specifically designed to introduce IMGs to Scotland's unique linguistic environment, underscoring a gap in the available support systems [6].

Barriers in communication due to different languages are a major risk to patient safety and quality of care, as it is widely acknowledged that effective communication between healthcare providers is essential [7]. On the other hand, better health outcomes have been associated with successful communication [8]. Many instances show serious mistakes caused by communication failures, like a Hispanic man misinterpreting “once” as “eleven” and a Chinese lady giving the wrong dose because of confusion about utensil measurements [9]. Doctors display fewer effective behaviours when communicating with patients from ethnic minorities [10]. Experience from the United States suggests that IMGs invest significant time in adapting to new languages, slang, and accents, which takes away their focus on patient care. Training programs should be created at an early stage to assist IMGs in navigating these challenges more effectively by using techniques like using non-verbal and written signals, adjusting their communication styles, and reinforcing information to patients [11]. Likewise, research has highlighted the significance of immigrants' familiarity with informal English and regional accents in aiding IMGs, mirroring concerns found in Australian studies [12-14].

In this review, the authors aim to tackle this issue by exploring Scotland's linguistic environment. They reviewed existing literature and held a focus group interview with IMGs in Scotland, discussing their encounters with Scottish languages and the impact on their clinical practice. One of the authors, himself an IMG, actively noted unfamiliar phrases and expressions used by patients and colleagues. The connotations of these terms were then analysed. The authors ultimately created a list of frequently used phrases and colloquialisms encountered in clinical settings during exchanges with patients as well as colleagues.

## Review

An overview of Scottish languages

Background on Scotland's Culture, History, and Linguistic Diversity

Countries can demonstrate their presence and uniqueness through the use of language [15]. Shared language promotes community, belonging, and civic engagement, facilitated by culture and education [16]. Language-based activism in Scotland has impacted Scottish nationalism [17]. This can be seen in the dual usage of Scottish and Gaelic on signs at train stations, motorways, and official establishments like the Scottish Parliament. Some claim that this is an example of hackneyed nationalism [18]. Even though opinions vary, language is essential in shaping Scottish national identity and culture [19]. Despite the significance of its languages in promoting nationalism, Scotland possesses a diverse literary heritage, intricate linguistic past, and overlapping language variations [20]. Here is a quick summary of the linguistic environment in Scotland. The linguistic landscape is the term used to refer to “the visibility and salience of languages on public and commercial signs” [21].

The usage of Gaelic is often associated with conveying power and status among certain groups and communities [21,22]. Heritage languages like Gaelic are considered public goods, meaning their use by one person doesn't reduce their availability to others [23]. Although just 1.7% of Scotland's populace speaks Gaelic fluently, primarily in the Highlands and Islands [24], the language is gaining traction, even in urban centres like Edinburgh and Glasgow [25]. Nearly 40% of people see Gaelic as an important part of their national identity [24]. In spite of the drop in Gaelic speakers, the language is expected to remain a key part of Scotland's national identity [26]. Many adults who do not speak Gaelic express a desire to learn it, viewing it as a cultural emblem [27]. Additionally, Gaelic and Celtic influences are present in Scots, with words like “smashing” originating from the Gaelic “smasheen”. Efforts to promote Gaelic, such as dedicated TV channels and bilingual signage, have been successful in maintaining the language's presence in Scotland [28].

Scots, which is basically the Scottish language, drifted apart from English after being isolated from “Inglis” following the Norman Conquest. Geographical factors and the Normans' disinterest in politics influenced this linguistic difference. In contemporary society, around 28% of people in Scotland speak Scots, frequently blending it with English [24]. After the Jacobite Rebellion of 1745, Scots experienced an increasing feeling of being inferior, which led to the prohibition of the kilt during that time. In the past, Scots was commonly used in the Church and Law in Scotland, but its popularity diminished greatly during the 19th century. Rabbie Burns (1759-1796) was a well-known supporter of the Scottish language and culture.

Scots has evolved through influences from various linguistic sources. The Anglian tribe, a Germanic community that arrived in the 6th century, laid early foundations for the language. Scandinavian elements were introduced through trade with Nordic countries and the Hanseatic League. In 1468, the King of Norway pledged Orkney as collateral for his daughter's dowry, who was set to marry James III of Scotland; Shetland was also transferred to the Scottish Crown that same year. Additionally, the French language significantly impacted Scots during the reign of Mary, Queen of Scots (1542-1567) because of the longstanding Auld Alliance between France and Scotland [24].

Individuals from Scotland also played significant roles in governing and expanding the British Empire, contributing substantially to its administration and growth. The status and importance of Scots in Scotland's linguistic landscape is a topic of continued discussion. Some contend that Scots is a separate language because of its distinct grammar and pronunciation, while others view it as one of Scotland's languages along with Gaelic. References to court usage pre-1707 and its link to famous poet Robert Burns reinforce the argument for Scots as a separate language. Despite some disagreement, Scots is widely embraced in Scotland, serving as a symbol of the nation's identity. It is even referred to as the language of the hearts by some [29].

The importance of language in effective patient care

Language is a crucial component in healthcare, functioning as the main channel for healthcare professionals to communicate with patients, comprehend their issues, and deliver proper treatment. Clear communication in healthcare is closely tied to patient outcomes, as it facilitates precise diagnosis, treatment compliance, and overall patient satisfaction [7]. When language barriers are present, they can result in misunderstandings, incorrect diagnoses, and potentially dangerous medical errors [30]. This is especially important in diverse and multilingual environments, where patients may use different languages or dialects than their healthcare providers.

In the NHS setting, where IMGs have a major impact, it is essential to have a high level of proficiency in English. Yet, fluency in English is not enough; healthcare professionals need to understand the subtle language variations and cultural backgrounds in their practice settings [31]. This involves comprehending local accents, informal words, and cultural phrases that are distinct to certain regions, like Scotland. Lacking linguistic and cultural competence could make it challenging for IMGs to establish trust with patients, a crucial aspect of providing quality patient care [32].

The relevance of learning Scottish colloquialisms to foster doctor-patient relationships and build rapport with colleagues

In Scotland, as in many places with unique linguistic traditions, local dialects and colloquialisms are deeply woven into day-to-day communication. For IMGs in the Scottish NHS, knowing Scottish colloquialisms is crucial for building effective doctor-patient relationships beyond mere language skills. Patients frequently use local dialects and expressions to describe their symptoms, voice concerns, or share emotions [33]. If healthcare providers are unfamiliar with these linguistic subtleties, it can lead to misunderstandings and obscurities in the patient’s account, potentially affecting the quality of care.

Furthermore, knowing and utilising Scottish colloquialisms can greatly improve an IMG’s connection with patients and co-workers. Establishing a connection is crucial in healthcare to earn patients’ trust, leading to open communication and compliance with medical recommendations [34]. Using familiar local phrases with co-workers can help improve communication and create a feeling of closeness and inclusion, which are crucial for effective teamwork and cooperation in the NHS [35]. Hence, it is essential for IMGs in Scotland to incorporate Scottish colloquialisms into their daily routine to effectively integrate into the NHS.

Common Scottish colloquialisms in daily doctor-patient communication

Table 1 presents commonly used Scottish colloquialisms for greetings and casual conversation, while Table 2 highlights those frequently used by patients during history taking and physical exams.

**Table 1:** Commonly used Scottish colloquialisms for greetings and general talk.

Domains	Scottish phrase	Meaning	Region	Reference
Greetings	"Aye, what's the craic?"	"Yes, what's happening?"	All of Scotland	[36]
	"How ye daein'?"	"How are you doing?"	All of Scotland	[37]
	"Haud yir weesht!"	"Be quiet!"	Central Scotland, Glasgow	[38]
	"Fit like?"	"How are you?"	North-East Scotland (Aberdeenshire)	[39]
	"Far a ye fae?"	"Where are you from?"	North-East Scotland (Aberdeenshire)	[39]
Expression of agreement	"Aye, that's braw!"	"Yes, that's great!"	All of Scotland	[36]
	"Och aye, ye're right enough!"	"Oh yes, you're quite right!"	All of Scotland	[37]
	"That'll dae nicely!"	"That will do nicely!"	All of Scotland	[39]
	"Aye, that's pure dead brilliant!"	"Yes, that's really excellent!"	All of Scotland, particularly Glasgow	[38]
	"On you go"	"Go ahead"	All of Scotland	[36]
Weather-related expressions	"Dreich"	"Dull, gloomy, or dreary weather"	All of Scotland	[37]
	"Scorchio"	"Very hot"	All of Scotland	[39]
	"Fower seasons in a day"	"Experiencing multiple types of weather in one day"	All of Scotland	[38]
Food and drink	"Tablet"	"A type of Scottish sweet, similar to fudge"	All of Scotland	[36]
	"Haggis"	"Traditional Scottish dish made of sheep's offal"	All of Scotland	[37]
	"Neeps and tatties"	"Turnips and potatoes"	All of Scotland	[39]
	“Mince and tatties”	"Minced beef and potatoes"	All of Scotland	[38]
	"A wee dram"	"A small measure of whisky"	All of Scotland	[36]
Miscellaneous	"Tuppence"	"Two pence"	All of Scotland	[37]
	"Cut it fine"	"Leave very little time to complete something"	All of Scotland	[39]
	"Borrow your ear"	"Ask for your attention"	All of Scotland	[38]
	"Doing the sweep"	"Checking all areas carefully"	All of Scotland	[36]
	"Haud yer weisht"	"Be quiet"	Central Scotland, Glasgow	[37]
	"Off you pop"	"You can go now"	All of Scotland	[39]
	"Crack on"	"Continue with something"	All of Scotland	[38]
	"Awfy thoctfu doac"	"Very thoughtful doctor"	Central Scotland, Glasgow	[36]
	"Awfy mensefu"	"Very polite or well-mannered"	Central Scotland, Glasgow	[37]
	"She diagnosed ma tribble in nae time"	"She diagnosed my trouble in no time"	All of Scotland	[39]
	"I ken/I dinnae ken"	"I know/I don't know"	All of Scotland	[38]
	"Guts for garters"	"Punishment is coming"	All of Scotland	[36]
	"Make headway"	"Make progress"	All of Scotland	[37]

**Table 2:** Common Scottish colloquialisms used by patients during history taking and physical examination.

Domains	Scottish phrase	Meaning	Region	Reference
Presenting complaint	"No awfy well"	"Not feeling very well"	All of Scotland	[39]
	"Awfy no well"	"Feeling very unwell"	All of Scotland	[38]
	"Having a sair heid"	"Having a headache"	All of Scotland	[36]
	"Scunnered"	"Fed up or annoyed"	All of Scotland	[37]
	"Stervin"	"Very hungry"	All of Scotland	[39]
	"I've got the lurgie"	"I've got an illness"	All of Scotland	[38]
	"Having a gippy tummy"	"Having an upset stomach"	All of Scotland	[36]
	"Wheesht"	"Be quiet"	Central Scotland, Glasgow	[37]
	"Drookit"	"Soaked to the skin"	All of Scotland	[39]
	"I'm as stiff as a board"	"Feeling very stiff"	All of Scotland	[38]
	"Crabbit"	"Grumpy or irritable"	All of Scotland	[36]
	"My feet puttin’"	"My feet are aching"	All of Scotland	[37]
	"Nay bad"	"Not bad"	All of Scotland	[39]
	"Groggy"	"Feeling weak or dazed"	All of Scotland	[38]
Past medical and surgical history	"Havin’ had auld bother"	"Having had old trouble"	All of Scotland	[36]
	"Been through the wars"	"Having had a difficult time"	All of Scotland	[37]
	"Had an MOT"	"Had a medical check-up"	All of Scotland	[39]
	"Recovered from the Muckle scunner"	"Recovered from a great annoyance or trouble"	All of Scotland	[38]
	"Been on the mend"	"Recovering"	All of Scotland	[36]
Drug history	"Been on the medicine merry-go-round"	"Trying many different medications"	All of Scotland	[37]
	"Been through the medicine cabinet"	"Used a lot of different medications"	All of Scotland	[39]
Social history	"Lived in a wee village"	"Lived in a small village"	All of Scotland	[38]
	"Worked in the shipyards"	"Worked in shipbuilding"	All of Scotland	[36]
	"Belonged to tight-knit community"	"Part of a close community"	All of Scotland	[37]
	"Grew up in the tenements"	"Grew up in apartment buildings"	All of Scotland	[39]
	"In the scheme"	"In a public housing area"	All of Scotland	[38]
	"Worked the land"	"Worked in agriculture"	All of Scotland	[36]
	"Worked the pits"	"Worked in coal mines"	All of Scotland	[37]
	"Rough"	"Tough or difficult"	All of Scotland	[39]
Family history, general exam	"Runs in the family"	"A common trait in the family"	All of Scotland	[38]
Observations	"Givin him a wee check"	"Giving him a quick check-up"	All of Scotland	[36]
General exam	"Peely-Wally"	"Pale or sickly looking"	All of Scotland	[37]
	"Hearty"	"Healthy and strong"	All of Scotland	[39]
	"Fousty"	"Musty or stale"	All of Scotland	[38]
Cardiovascular system	"Hert"	"Heart"	All of Scotland	[36]
Respiratory system	"Chesty"	"Having a chest infection"	All of Scotland	[37]
Respiratory system, nervous system	"Coughin' up blood"	"Coughing blood"	All of Scotland	[39]
	"Struggle to catch yer breeth"	"Difficulty breathing"	All of Scotland	[38]
	"Bust a blood vessel"	"Broke a blood vessel"	All of Scotland	[36]
Mental health	"Heart-sinkin"	"Disheartening"	All of Scotland	[37]
Mental health	"He’s a self-willed, obstinate little prat"	"He's very stubborn"	All of Scotland	[39]
	"Heid doacters"	"Psychiatrists or mental health doctors"	All of Scotland	[38]
	"Nutter"	"Someone who is mentally unstable"	All of Scotland	[36]
Gastrointestinal system	"Blawn up"	"Feeling bloated"	All of Scotland	[37]
Gastrointestinal system, Skin	"Cannae keep owt doon"	"Can't keep anything down"	All of Scotland	[39]
	"Got the runs"	"Diarrhea"	All of Scotland	[38]
	"Stomach bug"	"Stomach illness"	All of Scotland	[36]
	"Heavin"	"Vomiting"	All of Scotland	[37]
	"Spew"	"Vomit"	All of Scotland	[39]
	"Boaking"	"Throwing up"	All of Scotland	[38]
	"A wee spot"	"A small amount"	All of Scotland	[36]
Skin, musculoskeletal system	"Scaly"	"Covered in scales or rough patches"	All of Scotland	[37]
	"Ma skin’s affa dry"	"My skin is very dry"	All of Scotland	[39]
	"The midgies are eatin’ me alive"	"Being bitten by midges (small biting insects)"	All of Scotland, particularly the Highlands	[38]
	"Ma skin’s aw raw an’ sair"	"My skin is sore and raw"	All of Scotland	[36]
	"Ma back’s playing’ up"	"My back is causing trouble"	All of Scotland	[37]
Musculoskeletal system, obstetrics and gynaecology	"Ma knee’s gien’ me jip"	"My knee is giving me trouble"	All of Scotland	[39]
	"I've pulled a muscle in ma shoulder"	"I've injured a muscle in my shoulder"	All of Scotland	[38]
	"Ma elbow gubbed"	"My elbow is hurt"	All of Scotland	[36]
	"Ma joints are fair creekin"	"My joints are very stiff or sore"	All of Scotland	[37]
	"Bahoochie"	"Buttocks"	All of Scotland	[39]
	"I am expecting a wee bairn"	"I am expecting a baby"	All of Scotland	[38]
Obstetrics and gynaecology, paediatrics	"The bairn’s due any day noo"	"The baby is due any day now"	All of Scotland	[36]
	"The wean’s in the breech position"	"The baby is in the breech position (feet first)"	All of Scotland	[37]
	"Its time tae push"	"It's time to deliver the baby"	All of Scotland	[39]
	"Wee yin’s due vaccination"	"The baby is due for a vaccination"	All of Scotland	[38]
Paediatrics investigations	"Ma bairn’s got a sair belly"	"My child has a sore stomach"	All of Scotland	[36]
	"The wee yin’s got a braw smile"	"The baby has a beautiful smile"	All of Scotland	[37]
	"Bairn’s hospital"	"The child's hospital"	All of Scotland	[39]
	"The wean’s growin up so fast"	"The baby is growing up quickly"	All of Scotland	[38]
	"I am like a pin cushion"	"I have many injections or punctures"	All of Scotland	[36]
Diagnosis & treatments	"Ye've got auld man's troubles"	"You have typical old age problems"	All of Scotland	[37]
	"Ye've been hit wi' the glaikit bug"	"You've been struck with a silly problem"	All of Scotland	[39]
	"Ye've been dealt a dunt."	"You've been hit or injured"	All of Scotland	[38]
	"We'll get ye sorted oot"	"We'll get you sorted out"	All of Scotland	[36]
	"Work the magic"	"Perform a miracle or fix something impressively"	All of Scotland	[37]

Practical applications in healthcare settings

The Relevance of Scottish Colloquialisms in Healthcare Environments

In healthcare, language is essential for clear, compassionate, and effective communication between providers and patients. Scottish colloquialisms, deeply embedded in local culture, play a significant role in shaping communication dynamics within Scottish healthcare settings. These expressions go beyond informal language; they convey cultural meanings and subtleties that are vital for patients in expressing their emotions, experiences and health concerns [33]. IMGs working in the NHS often face challenges adapting to local language nuances. Understanding colloquial expressions not only helps in better communication but also strengthens the trust and connection between doctors and patients, which is crucial in building rapport and ensuring effective care. Studies suggest that adapting to regional dialects or colloquialisms enhances patient-centred communication, fostering trust and improving overall healthcare delivery [40].

Additionally, incorporating local dialects and colloquialisms by healthcare professionals can aid in establishing a culturally sensitive care setting, known to be crucial in lessening healthcare disparities and enhancing patient results [41]. Using language that reflects the patient's cultural background can help them feel heard and valued, leading to a more welcoming and compassionate healthcare environment [30].

Using Colloquialisms to Facilitate Doctor-Patient Communication, Alleviate Anxiety, and Improve Patient Satisfaction

Healthcare providers who strategically use Scottish colloquialisms can enhance communication by bridging linguistic gaps and making interactions feel more relatable and less daunting for patients. For many, particularly older adults or those from rural areas, hearing familiar language during medical consultations can ease anxiety and foster a sense of comfort [42]. This becomes especially crucial in high-stress situations where patients may already feel vulnerable; when doctors use familiar expressions, it can reassure patients that they are in capable hands, ultimately boosting their overall satisfaction with the care they get [43].

Furthermore, studies have indicated that patients are more inclined to have open conversations, inquire, and share their worries when they feel that their healthcare providers understand their language, both literally and figuratively [34]. This level of communication is essential for precise diagnosis, efficient treatment planning, and making sure patients follow prescribed treatments. Hence, incorporating colloquial language can have a significant impact on enhancing communication and decreasing the emotional gap between healthcare provider and patient, thus leading to better care quality and patient outcomes [44].

Examples of Scenarios Where Appropriate Use of Colloquialisms May Enhance Doctor-Patient Communication

In practice, incorporating Scottish colloquialisms into doctor-patient interactions can significantly improve communication. For example, when a patient from a rural area describes symptoms of a common illness, using local terms can make the patient feel more understood and valued. A doctor might say, “You’re feeling a bit peely-wally today”, a Scottish phrase meaning unwell or out of sorts. This kind of language use can put the patient at ease, reinforcing that the doctor is sensitive to their cultural and linguistic context [45].

In clinical settings, particularly with senior patients, the use of familiar language can have a comforting effect. For example, phrases like "nae bother", which translates to "no problem" in Scots, can significantly reduce a patient's anxiety. This aligns with the notion that patient reassurance and trust are enhanced when clinicians align their communication to the patient's cultural and linguistic expectations [40].

Additionally, when discussing sensitive subjects like end-of-life care, using colloquial language can ease the seriousness of the conversation, making it less daunting for patients and their families to participate [46]. For instance, a doctor might say, “Let’s have a blether about what’s next”, with “blether” being a Scottish term for “chat”, signalling a more casual and open dialogue. This technique can help simplify complex medical topics, making them more approachable for patients and enhancing their comprehension and acceptance of the care process [42].

The Scottish Government is dedicated to enhancing international workforce recruitment in health and social care. This initiative includes building a strong infrastructure within every health board to provide top-tier support for incoming international staff and boost the global recognition of the NHS Scotland brand [47]. As part of this effort, the government has allocated £11 million for new nationally coordinated international recruitment campaigns and the creation of a national Centre for Workforce Supply (CWS) [48]. The CWS will oversee annual recruitment drives to achieve the projected 1% net workforce growth needed for the 2022-2026 NHS Recovery Plan. Additionally, the government is providing £1 million in current-year funding to territorial health boards, enabling each board to appoint an international recruitment lead, with ongoing annual funding of £1 million to support these positions. The funding will be utilised to offer assistance to global employees, including providing objective structured clinical examination (OSCE) training to facilitate obtaining regulatory body registration [47]. The Scottish Government has set up the NHS Scotland Academy, in collaboration with NHS Golden Jubilee and NHS Education for Scotland, to provide fast-track training for various health and social care positions [48]. At the moment, the academy is in charge of creating and providing various personalised training options in fields such as diagnostics, pharmacy, peri-operative practice, and anaesthetics, along with specialised assistance for internationally hired staff.

Scottish health boards have hired over 1,000 healthcare support staff and nearly 200 nurses from abroad to help tackle the significant challenges facing the NHS [49]. Launched by Health Secretary Humza Yousaf last October, the support staff recruitment campaign has placed new employees in various roles within acute hospitals and community health teams. The international nurse recruitment initiative has resulted in 191 nurses, primarily from India and the Philippines, accepting job offers, with some already working in hospitals across Scotland. Agreements with recruitment agencies are also in place to bring in an additional 203 nurses. This number is expected to grow considerably in the coming months as health boards utilise new infrastructure for hiring qualified international staff. All recruitment follows the Scottish Code of Practice for health and social care personnel.

## Conclusions

It is essential for IMGs in Scotland to grasp and utilize Scottish slang as it improves communication and care between doctors and patients by mirroring the cultural setting. Developing the ability to effectively communicate in language is vital for successful healthcare provision and collaboration. Immersing in Scottish culture during the holiday season helps IMGs with their social and professional integration. This immersion in different cultures improves relationships with patients and strengthens team dynamics through understanding the social factors that impact healthcare. Effective communication is crucial for enhancing patient outcomes and professional relationships, requiring proficiency in local colloquialisms. This skill improves patient satisfaction, treatment adherence, and teamwork among healthcare teams for IMGs.
